# Global Proteomic Analyses Reveals Abnormal Immune Regulation in Patients With New Onset Ankylosing Spondylitis

**DOI:** 10.3389/fimmu.2022.838891

**Published:** 2022-03-17

**Authors:** Zongchao Yu, Xiaoping Hong, Xiaoli Zhang, Fengping Zheng, Fanna Liu, Huixuan Xu, Chengxin Zhu, Wanxia Cai, Dongzhou Liu, Lianghong Yin, Bo Hu, Donge Tang, Yong Dai

**Affiliations:** ^1^ Department of Nephrology, The First Affiliated Hospital of Jinan University, Guangzhou, China; ^2^ Guangdong Provincial Engineering Research Center of Autoimmune Disease Precision Medicine, The First Affiliated Hospital of Southern University of Science and Technology, Shenzhen People’s Hospital, The Second Clinical Medical College of Jinan University, Shenzhen, China; ^3^ Fifth Department of Medicine (Nephrology/Endocrinology/Rheumatology), University Medical Centre Mannheim, University of Heidelberg, Heidelberg, Germany

**Keywords:** ankylosing spondylitis (AS), global proteomic, immune regulation, peripheral blood mononuclear cells (PBMC), phosphorylation

## Abstract

**Background:**

Ankylosing spondylitis (AS) is a chronic inflammatory disease with serious consequences and a high rate of morbidity and mortality, In our previous work, we reveal the key features of proteins in new-onset ankylosing spondylitis patients.

**Material and Methods:**

Ankylosing spondylitis (AS) is a chronic inflammatory condition that affects the spine, and inflammation plays an essential role in AS pathogenesis. The inflammatory process in AS, however, is still poorly understood due to its intricacy. Systematic proteomic and phosphorylation analyses of peripheral blood mononuclear cells (PBMCs) were used to investigate potential pathways involved in AS pathogenesis.

**Results:**

Liquid chromatography-tandem mass spectrometry (LC–MS/MS) analysis was performed and discovered 782 differentially expressed proteins (DEPs) and 122 differentially phosphorylated proteins (DPPs) between 9 new-onset AS patients and 9 healthy controls. The DEPs were further verified using parallel reaction monitoring (PRM) analysis. PRM analysis verified that 3 proteins (HSP90AB1, HSP90AA1 and HSPA8) in the antigen processing and presentation pathway, 6 proteins (including ITPR1, MYLK and STIM1) in the platelet activation pathway and 10 proteins (including MYL12A, MYL9 and ROCK2) in the leukocyte transendothelial migration pathway were highly expressed in the PBMCs of AS patients.

**Conclusion:**

The key proteins involved in antigen processing and presentation, platelet activation and leukocyte transendothelial migration revealed abnormal immune regulation in patients with new-onset AS. These proteins might be used as candidate markers for AS diagnosis and new therapeutic targets, as well as elucidating the pathophysiology of AS.

## Introduction

Ankylosing spondylitis (AS) is a chronic inflammatory disease that can cause significant complications and is associated with high morbidity and mortality (1–3). The pathologic mechanism of AS remains largely unclear but is thought to be immune-mediated and has a strong genetic association with HLA-B27, a class I human leukocyte antigen (4, 5). Studies on AS have revealed that there is a tendency for endothelial dysfunction (6), and inflammation plays an important role in endothelial dysfunction (7). However, the complexity of the inflammatory process in AS is still poorly understood. Platelets have a major role in the interplay among atherogenesis, thrombosis, and inflammation (8). The interaction of platelets with leucocytes, their ability to modify leucocyte functions, their adherence to the endothelium, and their ability to stimulate endothelial cells are all significant functions of platelets (9). There is evidence suggesting that platelet activation may be an indicator of AS progression (10).

Proteomics is a new technology that identifies protein networks using a high-throughput discovery approach with high sensitivity and accuracy (11, 12). Phosphorylation of proteins is one of the most fundamental, widespread, and critical methods for regulating protein activation and function (13). Abnormal phosphorylation is associated with many diseases, and it is an indicator of abnormal status of organisms (14). However, There hasn’t been a systematic proteome and phosphorylation analysis of a particular cell type in AS yet. Professional antigen-presenting cells, such as macrophages and dendritic cells, are derived from monocytes, and the role of monocytes in AS is still elusive (15).

In the present study, we used proteomic and phosphomic techniques coupled with systematic bioinformatics analysis to show that differences in protein expression and phosphorylation in AS peripheral blood mononuclear cells (PBMCs) implicate potential pathways involved in disease pathogenesis. We used PRM to evaluate target proteins to obtain a more global picture of protein expression in AS patients.

## Patients and Methods

### Patients

The clinical information of the participants has been reported in our previous study (16). This observational study was conducted at the Department of Nephrology, The Affiliated Hospital of Jinan University (Guangzhou, China). All procedures were approved by the local ethical committee of The Affiliated Hospital of Jinan University and followed the tenets of the Declaration of Helsinki. Nine new-onset HLA-B27-positive AS patients who fulfilled the 1984 revised New York criteria (17) and nine age-matched healthy controls were recruited [median age (interquartile range (IQR), 27 (30, 34) vs. 34 (29.5, 36), respectively]. Patients with autoimmune diseases and infectious diseases were excluded from this study. Cubital vein blood samples were collected after obtaining informed consent from the participants. An automated blood cell counter was used to determine the white blood cell count, lymphocyte, monocyte, and neutrophil differentials throughout a routine blood examination. (Beckman Coulter, Ireland).

### Isolation of PBMCs

Peripheral venous blood (10 ml) was collected from each individual in ethylenediaminetetraacetic acid (EDTA) anticoagulant tubes and diluted with an equal volume of phosphate-buffered saline (PBS). Diluted blood was gently overlaid on 10 ml of Ficoll-Paque Plus solution (Amersham Biosciences, Uppsala, Sweden) and centrifuged at 500 g for 20 min at room temperature. PBMCs were collected from the interphase and washed twice with PBS. The supernatant was discarded, and the PBMCs were then resuspended at a final concentration of 1 × 10^6^ cells/ml in RPMI 1640 medium (Gibco) containing 10% heat-inactivated fetal calf serum, 100 U/mL penicillin and 100 µg/mL streptomycin. The viability of the cells was consistently greater than 95% as measured by trypan blue solution (Sigma, Germany).

### Protein Extraction and Trypsin Digestion

The protein extraction and digestion procedures were performed according to a previously published method (18). We removed samples from -80°C cryogenic storage and then sonicated them three times on ice using a high-intensity ultrasonic processor (JY92-IIN, Scientz, China) in a fourfold volume of lysis buffer (containing 8 M urea, HALT protease and phosphatase inhibitor cocktail). The cell fragments were removed by centrifugation for 10 min at 12 000 g at 4°C. Finally, the supernatant was collected and transferred to a new centrifuge tube. The protein concentration was determined using a BCA kit (Beyotime, China).

For digestion, the protein solution was reduced with 5 mM dithiothreitol (DTT) for 30 min at 56°C and alkylated with 11 mM iodoacetamide (IAA) for 15 min at room temperature in darkness. Protein samples were diluted to less than 2 M by adding 100 mM triethyl ammonium bicarbonate (TEAB), and trypsin (Promega, USA) was added at a 1:50 trypsin-to-protein mass ratio for the first digestion overnight followed by a second 1:100 trypsin-to-protein digestion for 4 h.

### Modification Enrichment and Affinity Enrichment

For the enrichment of the modified peptides, peptide mixtures were incubated with IMAC microsphere suspensions with vibration in loading buffer (50% acetonitrile/6% trifluoroacetic acid). The IMAC microspheres with enriched phosphopeptides were collected by centrifugation, and the supernatant was removed. The IMAC microspheres were then sequentially washed with 50% acetonitrile/6% trifluoroacetic acid and 30% acetonitrile/0.1% trifluoroacetic acid. The enriched phosphopeptides were eluted from the IMAC microspheres with elution buffer containing 10% NH4OH. Finally, the eluted phosphopeptides were combined and vacuum dried. For LC–MS/MS analysis, the resulting peptides were desalted with C18 ZipTips (Millipore) according to the manufacturer’s instructions.

### LC–MS/MS Analysis

We performed LC–MS/MS analysis on an EASY-nLC 1000 UPLC system (Thermo Fisher Scientific). Peptides were subjected to a NSI source followed by tandem mass spectrometry (MS/MS) in a Q Exactive Plus (Thermo Fisher Scientific) coupled online to the UPLC. Intact peptides were detected in the Orbitrap at a resolution of 70,000, and peptides were selected for MS/MS using a NCE setting of 28 and 30. Ion fragments were detected in the Orbitrap at a resolution of 17,500. A data-dependent procedure that alternated between one MS scan followed by 20 MS/MS scans was applied for the top 20 precursor ions above a threshold ion count of 5.0E3 in the MS survey scan with a 15.0 s dynamic exclusion. The applied electrospray voltage was 2.0 kV. Automatic gain control (AGC) was used to prevent overfilling of the orbitrap, and 5E4 ions were accumulated for the generation of MS/MS spectra. For MS scans, the m/z scan range was 350–1,800, and the fixed first mass was set as 100 m/z.

### Database Search and Bioinformatics Analysis

The MS/MS data were retrieved by the MaxQuant search engine (v1.6.6.0). A human database was searched (Swiss-Prot, downloaded on May 13, 2019), retrieving 20422 protein sequences. The decoy database anti-library was used to reduce the false-positive rate (FDR). FDR was adjusted to < 1%, and the minimum score for modified peptides was set > 40. The ratio of the average protein intensity between AS patient samples and healthy control samples was considered as the relative quantitative value. Proteins with a fold-change ≥1.50 or ≤0.67 were considered expression/phosphorylation significant. Based on the protein sequence alignment method, the protein domain functions were defined by InterProScan (http://www.ebi.ac.uk/interpro/). Functional annotation enrichments of differentially expressed proteins (DEPs) and differentially phosphorylated proteins (DPPs) were performed by Gene Ontology (GO) annotation analysis and Kyoto Encyclopedia of Genes and Genomes (KEGG) analysis. Significant enrichment was identified as *p* < 0.05 in Fisher’s exact test and q < 0.05 in Benjamini-Hochberg’s procedure. We conducted hierarchical clustering analysis for the DEPs and DPPs using the “heatmap.2” function form and “gplots” R package.

### Validation of Target Proteins Using Parallel Reaction Monitoring

The differential abundance of selected proteins was verified using PRM analysis. Skyline (v.3.6) software was used to process the resulting MS data. The parameters of the peptide segment were determined as follows: the enzyme was set at trypsin [KR/P]; the maximum number of missed cleavages was set as 2; and the length of the peptide was set as 8−25. The variable modification was set as carbamidomethyl on Cys and oxidation on Met, and the max variable modifications were set as 3. The transition parameters were as follows: the precursor charges were set as 2 and 3; the ion charges were set as 1 and 2; and the ion type was set as b, y and p. Fragment ion selection started from the third to the last, and the ion match tolerance was set as 0.02 Da.

## Results

The clinical and laboratory results between AS patients and healthy controls have been reported in our previous study (16).

### Identification of Protein Expression and Phosphorylation Changes

**Figure 1:**
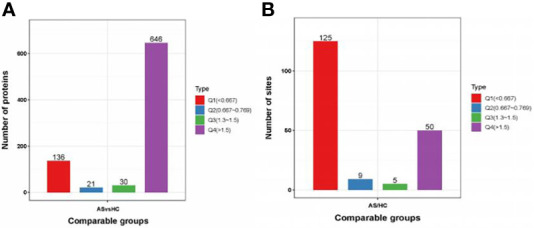
Protein identification. **(A, B)** DEPs and DPPs between AS patients and healthy controls. DEPs, differentially expressed proteins; DPPs, differentially Phosphorylated proteins.

A total of 2240 proteins were identified, and 782 proteins were significantly changed compared to the healthy controls. Of these altered proteins, 646 proteins were upregulated (≥1.5-fold), and 136 proteins were downregulated (≤0.67-fold) (**Figure 1A**). For protein phosphorylation, 210 phosphorylation sites in 122 proteins were significantly changed between AS patients and healthy controls. In addition, 50 phosphorylation sites were upregulated (≥1.5-fold) in 37 proteins, and 125 phosphorylation sites were downregulated (≤0.67-fold) in 85 proteins (**Figure 1B**).

### Peptide Motifs Associated With Phosphorylation

**Figure 2:**
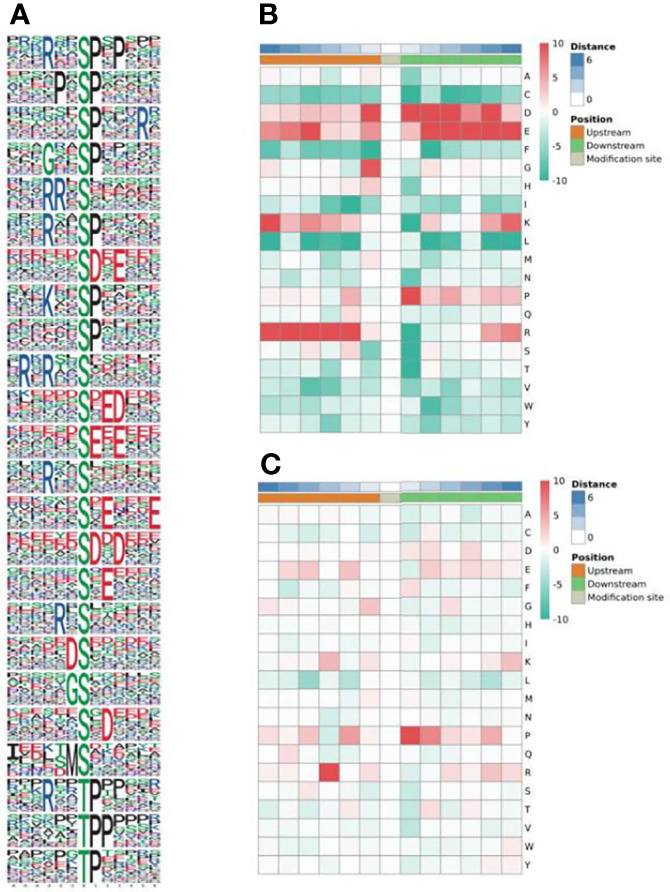
Enrichment analysis of phosphorylated protein motifs **(A)**. Heatmap of the amino acid compositions of the phosphorylation sites showing the frequency of the different types of amino acids around serine **(B)** and threonine **(C)**. Red indicates significant enrichment of the amino acid near the modification site, and green indicates significant reduction of the amino acid near the modification site.

In total, 1855 sequences, containing the 12 residues, were obtained with six upstream and six downstream residues surrounding each of the phosphorylation sites. Of these sequences, 1761 (94.93%) were centered on a serine residue, and 94 (5.07%) were centered on a threonine (**Figure 2A**). The serine phosphorylation category included 24 overrepresented motifs as follows: the most frequent motifs were ‘sP’ (291 occurrences) and ‘Rxxs’ (215 occurrences) followed by ‘PxsP’ (136 occurrences), ‘sDxE’ (119 occurrences) and ‘sxE’ (102 occurrences) (**Figure 2B**). ‘tP’ was the most frequent motif in the threonine phosphorylation category (**Figure 2C**).

### Annotation and Classification of All Identified DEPs and DPPs

**Figure 3:**
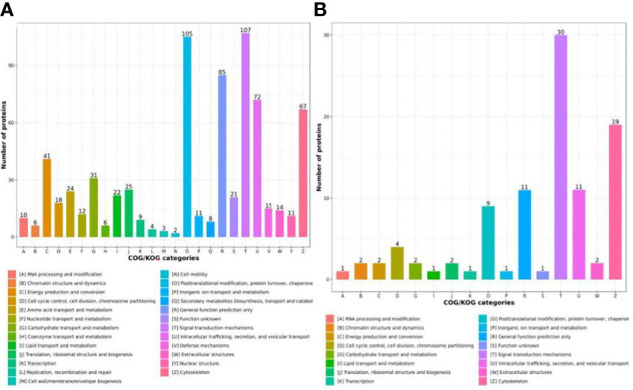
COG functional classification distribution map of DEPs **(A)** and DPPs **(B)**.

To investigate the functions of the identified and quantified proteins, we annotated functional category distributions according to COG categories. The differentially expressed proteins (DEPs) identified were classified into 25 COG categories. Among these, “Signal transduction mechanisms” (Group T, 107 DEPs) and “Posttranslational modification, protein turnover and chaperones “ (Group O, 105 DEPs) represented the two largest groups followed by “General function prediction only” (Group R, 85 DEPs), “Intracellular trafficking, secretion, and vesicular transport” (Group U, 72 DEPs) and “Cytoskeleton” (Group Z, 67 DEPs) (**Figure 3A**). Differentially phosphorylated proteins (DPPs) were classified into 16 COG categories, and the most predominant categories were “Signal transduction mechanisms” (Group T, 30 DPPs) and “Cytoskeleton” (Group Z, 19 DPPs) (**Figure 3B**).

### Enrichment Analysis of DEPs and DPPs

**Figure 4:**
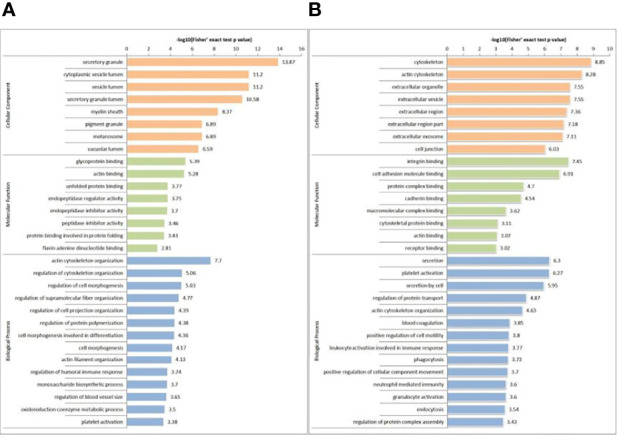
GO-based enrichment analysis of DEPs **(A)** and DPPs **(B)** in terms of cellular component, molecular function and biological process.

In GO terms, 30 different groups of pathways were established for DEPs and DPPs, which were further divided into the following three classifications: cellular component (n=8), molecular function (n=8) and biological process (n=14). For DEPs, the most enriched GO terms were as follows: ‘actin cytoskeleton organization’ (68 proteins) in biological process; ‘secretory granule’ (193 proteins) in cellular component; and ‘glycoprotein binding’ (23 proteins) in molecular function (**Figure 4A**). For DPPs, the most enriched GO terms were as follows: ‘secretion’ (34 proteins) in biological process; ‘cytoskeleton’ in cellular component (51 proteins); and ‘integrin binding’ (9 proteins) in molecular function (**Figure 4B**).

**Figure 5:**
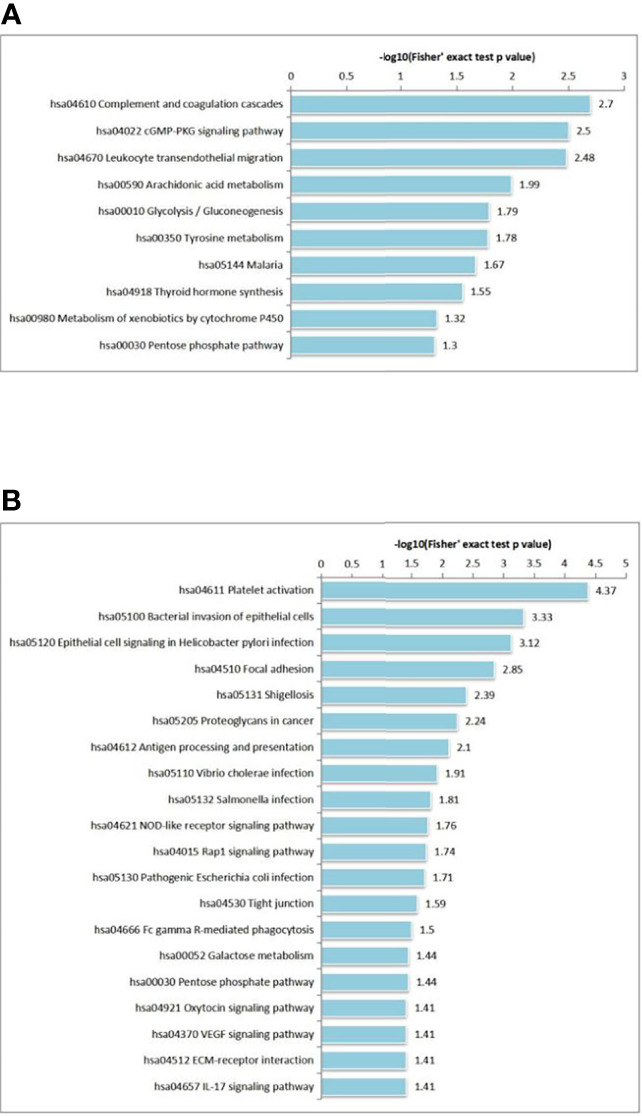
KEGG pathway-based enrichment analysis of DEPs **(A)** and DPPs **(B)**.

We also performed KEGG pathway enrichment analysis by calculating Fisher’s exact test P values to characterize the predicted roles of highly altered proteins. KEGG pathway-based enrichment analysis revealed 10 pathways for DEPs, including ‘complement and coagulation cascades’ (27 proteins), ‘cGMP-PKG signaling pathway’ (27 proteins) and ‘leukocyte transendothelial migration’ (26 proteins). (**Figure 5A**). Regarding phosphorylation, 20 pathways were identified for DPPs, and ‘platelet activation’ (15 proteins) was the most significant pathway for enrichment (**Figure 5B**).

**Figure 6:**
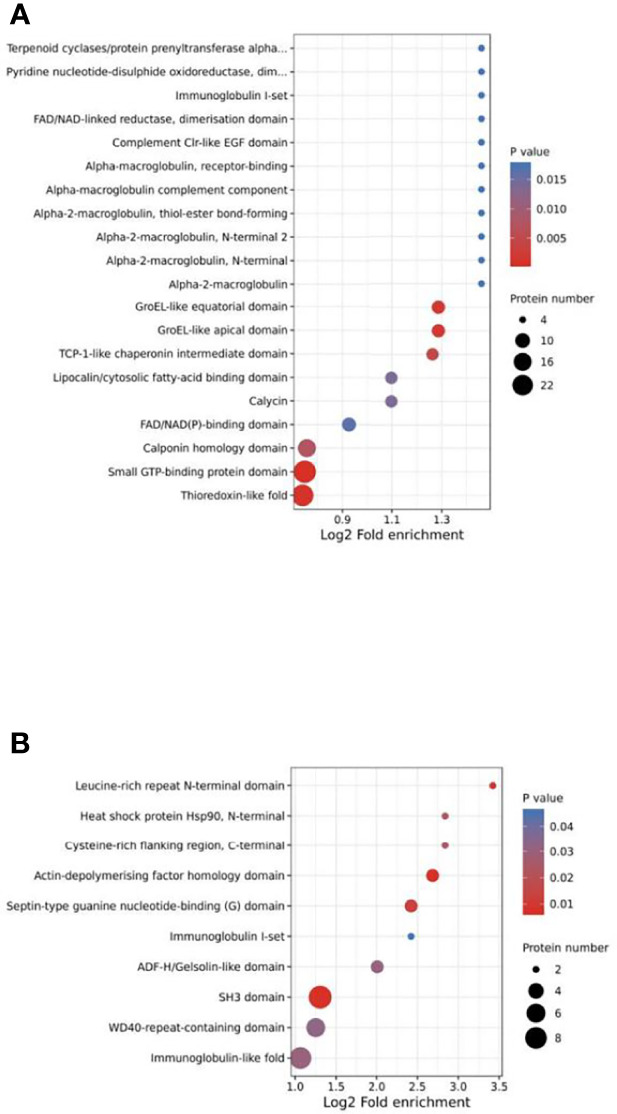
Protein domain enrichment analysis of DEPs **(A)** and DPPs **(B)**.

Protein domain enrichment analysis revealed that DEPs were significantly enriched in 25 terms, including small GTP-binding protein domain, thioredoxin-like fold, GroEL-like apical domain and GroEL-like equatorial domain. (**Figure 6A**). DPPs were significantly enriched in 10 terms, including actin-depolymerizing factor homology domain, SH3 domain and leucine-rich repeat N-terminal domain (**Figure 6B**).

### Protein–Protein Interaction Network Analysis

**Figure 7:**
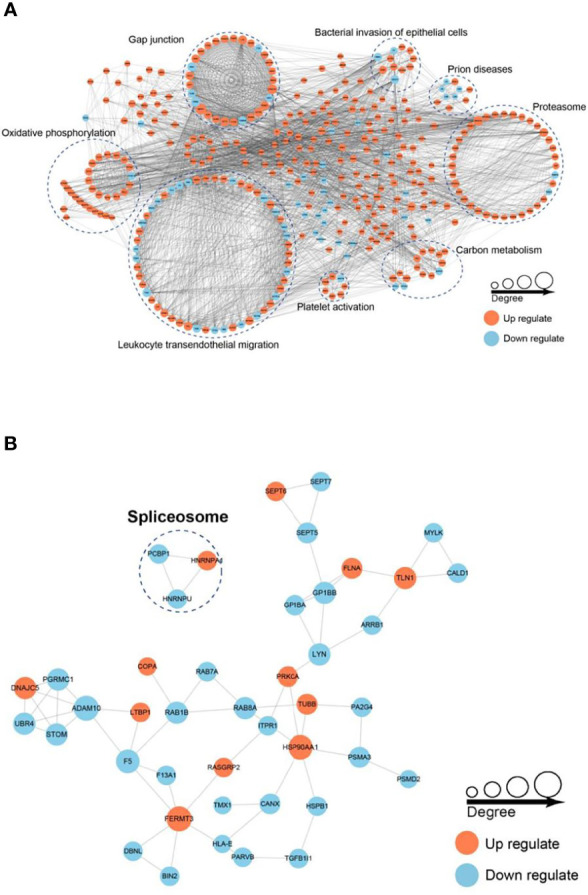
Protein–protein interaction (PPI) network analysis of DEPs **(A)** and DPPs **(B)**. The STRING database was used to annotate the functional interactions of all the identified DEPs and DPPs.

To better understand the interaction of the DEPs and DPPs, we subsequently constructed a PPI network using STRING software. A total of 782 DEPs and 43 DPPs were mapped to the protein interaction database, presenting a global view of the diverse cellular functions of DEPs and DPPs in AS. As shown in **Figure 7**, DEPs were involved in leukocyte transendothelial migration, oxidative phosphorylation, gap junctions, bacterial invasion of epithelial cells, prion diseases, proteasome, carbon metabolism and platelet activation, and DPPs were involved in spliceosome, comprising a dense protein interaction network. The physiological interactions among these protein complexes likely contribute to the cooperation and coordination of their functions in the control of numerous intracellular signaling and regulatory pathways in AS.

### Verification Results of Target Proteins

PRM for targeted quantitative proteins by MS was used to quantitatively identify target proteins. A total of 19 proteins were quantitatively identified (**Table 1**). These proteins were selected for their functional significance concluded from proteome analysis, and the ratio of protein abundance varied in a wide range. Among them, 3 proteins (HSP90AB1, HSP90AA1 and HSPA8) were involved in the antigen processing and presentation pathway, of which the highest differential expression was HSP90AA1 with an AS/HC ratio of 35.40. Six proteins (including ITPR1, MYLK and STIM1) were involved in the platelet activation pathway, of which the highest differential expression was observed for STIM1 with an AS/HC ratio of 22.35. Ten proteins (including MYL12A, MYL9 and ROCK2) were involved in the leukocyte transendothelial migration pathway, of which the highest differential expression was MMP9 with an AS/HC ratio of 18.97. The fold-change values for these proteins according to the PRM results are presented in **Table 1**. The PRM and label-free quantification (LQ) results were highly consistent.

**Table 1:** Verification results of target proteins.

KEGG pathway	Protein Accession	Gene	Protein description	AS Relative Abundence	HC Relative Abundence	Fold change (AS/HC Ratio) in PRM	Fold change (AS/HC Ratio) in LQ
hsa04612 Antigen processing and presentation	P08238	HSP90AB1	Heat shock protein HSP 90-beta	1.94	0.06	32.89	12.33
	P07900	HSP90AA1	Heat shock protein HSP 90-alpha	1.95	0.05	35.40	4.99
	P11142	HSPA8	Heat shock cognate 71 kDa protein	1.57	0.43	3.63	1.93
hsa04611 Platelet activation	Q14643	ITPR1	Inositol 1,4,5-trisphosphate receptor type 1	1.77	0.23	7.70	9.47
	Q15746	MYLK	Myosin light chain kinase, smooth muscle	1.91	0.09	21.84	8.35
	Q13586	STIM1	Stromal interaction molecule 1	1.91	0.09	22.35	6.07
	P07948	LYN	Tyrosine-protein kinase Lyn	1.80	0.20	8.79	3.20
	Q16539	MAPK14	Mitogen-activated protein kinase 14	1.20	0.80	1.50	2.68
	P12931	SRC	Proto-oncogene tyrosine-protein kinase Src	1.84	0.16	11.59	1.59
hsa04670 Leukocyte transendothelial migration	P19105	MYL12A	Myosin regulatory light chain 12A	1.79	0.21	8.72	17.35
	P24844	MYL9	Myosin regulatory light polypeptide 9	1.81	0.19	9.66	8.48
	O75116	ROCK2	Rho-associated protein kinase 2	1.90	0.10	18.75	6.12
	O43707	ACTN4	Alpha-actinin-4	1.64	0.36	4.62	5.23
	P14780	MMP9	Matrix metalloproteinase-9	1.90	0.10	18.97	4.41
	P05771	PRKCB	Protein kinase C beta type	1.72	0.28	6.05	3.48
	P18206	VCL	Vinculin	1.66	0.34	4.85	3.28
	P12814	ACTN1	Alpha-actinin-1	1.78	0.22	7.98	2.82
	P16284	PECAM1	Platelet endothelial cell adhesion molecule	1.79	0.21	8.48	1.88
	P11215	ITGAM	Integrin alpha-M	1.28	0.72	1.78	1.85

## Discussion

AS is a chronic systemic inflammatory disorder that mainly affects the sacroiliac joint and spine (2). Due the position limitation of AS, studies of AS-related proteins have been mainly restricted to arthritogenic proteins (11, 19, 20). Additional potential proteins should be investigated to promote the understanding of the pathological mechanism of AS.

In the present study, we performed comprehensive proteomic profiling and PRM analysis of PBMCs obtained from patients with AS. We discovered 782 DEPs and 122 DPPs in AS patients compared to healthy controls. PRM analysis for 19 proteins was performed to further verify the expression levels of target proteins. As a result, 3 proteins (HSP90AB1, HSP90AA1 and HSPA8) involved in antigen processing and presentation, 6 proteins (including ITPR1, MYLK and STIM1) involved in platelet activation and 10 proteins (including MYL12A, MYL9 and ROCK2) involved in leukocyte transendothelial migration were validated to be overexpressed in the PBMCs of AS patients.

Chronic inflammation of the central axis joints is the most significant pathological alterationin in AS, and the disease might gradually extended to internal organs (21). The inflammatory process in AS is still poorly understood, making it difficult to track disease activity and choose treatment strategies. The body tightly regulates inflammation, which is associated with transient crossing of leukocytes through the blood vessel wall, a process known as transendothelial migration. (TEM) (22). The CD18 integrin family plays a crucial role in guiding leukocytes to inflammatory areas (23, 24). Kragstrup et al. (25) demonstrated that that patients with arthritis contain anti-inflammatory soluble CD18 (sCD18) complexes in their blood and synovial fluid. MMP-3 and MMP-9 are involved in CD18 shedding processes (26, 27). MMP family proteins play a role in arthritis pathogenesis (28). In particular, MMP3 is a protease that issecreted and synthesized by fibroblasts and chondrocytes in synovial joints, and it activates other MMPs, such as MMP1, MMP7 and MMP9 (29). In AS, both MMP-3 and MMP-9 serum concentrations have been linked to disease activity (30, 31). Our findings were supported by the discovery of MMP9, a previously studied AS-related protein.

In clinical practice, platelet counts generally increase during the active stage of AS (32, 33). However, there are few studies on platelet function, particularly the activation function of platelets. CD62P, a membrane glycoprotein, is expressed on the α-granule membrane of standstill platelets when they are activated, according to a previous study, while CD63, a lysosome membrane glycoprotein, is also expressed on the platelet membrane when platelets are activated. CD63 and CD62P are platelet activation indicators, with the latter serving as the gold standard (10). Another study discovered that AS patients have significantly higher levels of platelet-monocyte complex (PMC) and soluble CD40L (sCD40L) than the control group (34). In the present study, we identified 6 key proteins involved in platelet activation that were highly expressed in PBMCs of AS patients. Based on previous studies and our findings, platelet activation could indicate an aggravation of AS.

Antigen processing and presentation play a critical role in the pathophysiology of AS (35–37). Human leukocyte antigens (HLAs) are proteins encoded by genes of the human major histocompatibility complex (MHC). The expression of HLA-B27 is highly linked with the presence of AS (38, 39), and other genes related to AS have been discovered (40, 41). In our previous studies, we identified differences in the expression and phosphorylation of HLA-E, calnexin (CANX), and two heat-shock proteins (HSPs) (HSP90AA1 and HSP90AB1) in PBMCs of AS patients and healthy controls (16). In the present study, we further verified these proteins by PRM and demonstrated that HSP90AA1 and HSP90AB1 were highly expressed, indicating that HSP90AA1 and HSP90AB1 may be involved in the development of AS.

Numerous limitations should be addressed in this investigation. First, The present study was a cross-sectional investigation of a modest sample size. Second, despite the fact that thousands of proteins were detected, no more than 1000 of them have quantitative data on their expression and phosphorylation. Some key proteins may have been overlooked due to missing key information. Future research should delve deeper into these limiting factors.

In conclusion, between AS patients and healthy controls, quantitative proteomic studies revealed differently expressed and phosphorylated proteins.In the PBMCs of patients with AS, key proteins involved in antigen processing and presentation, platelet activation, and leukocyte transendothelial migration were shown to be highly elevated. These proteins could be served as candidate markers for AS diagnosis and new therapeutic targets, as well as elucidating the pathophysiology of AS.

## Conclusion

Liquid chromatography-tandem mass spectrometry (LC–MS/MS) analysis was performed and discovered 782 differentially expressed proteins (DEPs) and 122 differentially phosphorylated proteins (DPPs) between 9 new-onset AS patients and 9 healthy controls. The key proteins involved in antigen processing and presentation, platelet activation and leukocyte transendothelial migration revealed abnormal immune regulation in patients with new-onset AS. These proteins may further help reveal the pathogenesis of AS and serve as candidate markers for AS diagnosis and new treatment targets.

## Data Availability Statement

The datasets presented in this study can be found in online repositories. The names of the repositories and accession numbers can be found below: http://proteomecentral.proteomexchange.org/cgi/GetDataset?ID=PXD019667.

## Ethics Statement

All procedures were approved by the local ethical committee of The Affiliated Hospital of Jinan University and followed the tenets of the Declaration of Helsinki.

## Author Contributions

YD, BH, and DT designed the study. ZY and FZ generated and analysed the data. ZY and XH interpreted the data and wrote the manuscript. All the authors revised the manuscript for intellectual content and approved its final version to be published. BH is the guarantor of this work.

## Funding

This work was supported by the Science and Technology Plan of Shenzhen (No. JCYJ20170307095606266); Sanming Project of Medicine in Shenzhen, the group of Rheumatology and Immunology leaded by Xiaofeng Zeng of Peking Union Medical College Hospital and Dongzhou Liu in Shenzhen People’s Hospital (SYJY201704 and SYJY201705); the Key Research and Development Program of Guangdong Province (Grant No. 2019B020229001); Shenzhen Key Medical Discipline Construction Fund (No.SZXK011); Postdoctoral Fund of the First Affiliated Hospital, Jinan University (Grant No. 809001); Young Innovative Talents Project of General Colleges and Universities in Guangdong Province (Grant No. 2018KQNCX010) and GuangDong Basic and Applied Basic Research Foundation (Grant No. 2020A1515111209).

## Conflict of Interest

The authors declare that the research was conducted in the absence of any commercial or financial relationships that could be construed as a potential conflict of interest.

## Publisher’s Note

All claims expressed in this article are solely those of the authors and do not necessarily represent those of their affiliated organizations, or those of the publisher, the editors and the reviewers. Any product that may be evaluated in this article, or claim that may be made by its manufacturer, is not guaranteed or endorsed by the publisher.
